# HIV-1 protease inhibitor mutations affect the development of HIV-1 resistance to the maturation inhibitor bevirimat

**DOI:** 10.1186/1742-4690-8-70

**Published:** 2011-08-24

**Authors:** Axel Fun, Noortje M van Maarseveen, Jana Pokorná, Renée EM Maas, Pauline J Schipper, Jan Konvalinka, Monique Nijhuis

**Affiliations:** 1 Department of Virology, Medical Microbiology, University Medical Center Utrecht, The Netherlands; 2 Gilead Sciences and IOCB Research Center, Institute of Organic Chemistry and Biochemistry of the Academy of Sciences of the Czech Republic, Prague, Czech Republic

## Abstract

**Background:**

Maturation inhibitors are an experimental class of antiretrovirals that inhibit Human Immunodeficiency Virus (HIV) particle maturation, the structural rearrangement required to form infectious virus particles. This rearrangement is triggered by the ordered cleavage of the precursor Gag polyproteins into their functional counterparts by the viral enzyme protease. In contrast to protease inhibitors, maturation inhibitors impede particle maturation by targeting the substrate of protease (Gag) instead of the protease enzyme itself. Direct cross-resistance between protease and maturation inhibitors may seem unlikely, but the co-evolution of protease and its substrate, Gag, during protease inhibitor therapy, could potentially affect future maturation inhibitor therapy. Previous studies showed that there might also be an effect of protease inhibitor resistance mutations on the development of maturation inhibitor resistance, but the exact mechanism remains unclear. We used wild-type and protease inhibitor resistant viruses to determine the impact of protease inhibitor resistance mutations on the development of maturation inhibitor resistance.

**Results:**

Our resistance selection studies demonstrated that the resistance profiles for the maturation inhibitor bevirimat are more diverse for viruses with a mutated protease compared to viruses with a wild-type protease. Viral replication did not appear to be a major factor during emergence of bevirimat resistance. In all *in vitro *selections, one of four mutations was selected: Gag V362I, A364V, S368N or V370A. The impact of these mutations on maturation inhibitor resistance and viral replication was analyzed in different protease backgrounds. The data suggest that the protease background affects development of HIV-1 resistance to bevirimat and the replication profiles of bevirimat-selected HIV-1. The protease-dependent bevirimat resistance and replication levels can be explained by differences in CA/p2 cleavage processing by the different proteases.

**Conclusions:**

These findings highlight the complicated interactions between the viral protease and its substrate. By providing a better understanding of these interactions, we aim to help guide the development of second generation maturation inhibitors.

## Background

Maturation is an essential step in the life-cycle of human immunodeficiency virus type 1 (HIV-1). It is the transition of the immature, non-infectious virus particle to the mature and infectious virion and is triggered by the proteolytic cleavage of the precursor Gag (Pr55^Gag^) and GagPol (Pr160^GagPol^) polyproteins by the viral enzyme protease. Gag is cleaved into the structural proteins matrix (MA, p17), capsid (CA, p24) and nucleocapsid (NC, p7), p6 and two small spacer peptides (p1 and p2). This protease-mediated cleavage elicits the structural rearrangement that results in the dense conical core, characteristic of infectious HIV-1 particles.

Since immature particles are non-infectious, particle maturation is an excellent target for antiretroviral drugs. Protease inhibitors (PI) successfully inhibit viral replication by targeting the enzyme responsible for maturation and have played a major role in antiviral therapy since their introduction in 1995. So far, nine different PIs have been approved for clinical use. However, a high degree of cross-resistance between protease inhibitors limits the utility of these inhibitors if PI resistance emerges.

Maturation inhibitors are a new class of antiretrovirals that also impede particle maturation but do so by targeting the substrate of protease (Gag) instead of the protease enzyme itself. Therefore, direct cross-resistance between PIs and maturation inhibitors may seem unlikely. However during PI treatment, co-evolution of the viral protease and its substrate Gag is common, which may have an effect on the subsequent utility of maturation inhibitors [1-5]. Several maturation inhibitors are or have been in development including: bevirimat (BVM, Panacos PA-457, Myriad MPC-4326); PA1050040, which is a second generation maturation inhibitor from Panacos [6], based on bevirimat; two maturation inhibitors from Myriad Pharmaceuticals, Vivecon (MPC-9055)[7,8] and MPI-461359 [9]; PF-46396 [10] from Pfizer and several capsid assembly inhibitors including CAP-1 [11], CAI[12], and BI-257, BI-627 and BI-720 from Boehringer-Ingelheim[13]. Bevirimat was the first of these maturation inhibitors to go into clinical trials and inhibits HIV-1 replication by specifically blocking cleavage of CA from p2, one of the final (rate-limiting) steps in the Gag processing cascade. Incomplete processing of CA from CA-p2 (p25) results in unsuccessful particle maturation and, therefore, non-infectious virions [14]. The CA/p2 cleavage site (CS) has been identified as the bevirimat target region by Western-blotting and *in vitro *resistance selection studies [14,15]. Nonetheless, the mechanism of action of bevirimat is still poorly understood as the actual binding site of bevirimat has not been identified. Recently, it has been shown that, besides sterically blocking the CA/p2 junction, bevirimat may have a stabilizing effect on the immature Gag lattice. This indicates that bevirimat binds during assembly and must be incorporated to inhibit maturation, which offers an explanation why bevirimat is unable to prevent cleavage of free Gag in solution[16].

Initial *in vitro *selection studies identified bevirimat resistance mutations in the CA/p2 cleavage site at Gag positions 358, 363, 364 and 366 [15]. Phase 2b clinical studies demonstrated that baseline polymorphisms slightly downstream of the CA/p2 cleavage site (Gag aa 369, 370 and 371, known as the QVT-motif) also confer resistance [17,18].

We previously showed that bevirimat resistance mutations are more frequently observed in PI resistant but bevirimat naïve HIV-1 isolates, compared to PI and bevirimat treatment naïve isolates; and this was mainly attributed to an accumulation of mutations in the QVT-motif [19]. This study also showed that mutations associated with bevirimat resistance were detected more frequently in HIV-1 isolates with three or more PI resistance mutations than in those with less than three PI resistance mutations. Conversely, Adamson and colleagues suggested that mutations in the viral protease affecting viral replication may delay the selection of maturation inhibitor resistance [20].

To better understand the effect of PI therapy on viral susceptibility to maturation inhibitors, we set up a maturation inhibitor model system. We performed multiple *in vitro *selection studies with ten different viruses that contained PI resistance mutations in the viral protease and/or Gag CS and that displayed a broad range of replication capacities (RC). Subsequently, we conducted a detailed analysis of the identified resistance mutations. The data in this paper clearly demonstrate that PI resistance mutations alter the resistance profiles for the maturation inhibitor bevirimat. We also show that the protease background determines the level of maturation inhibitor resistance and viral replication.

## Results

### *In vitro *selections

**Figure 1:**
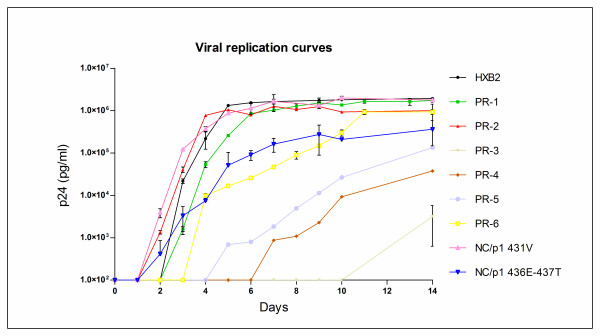
**Replication capacity of the ten viruses that were used for the *in vitro *selection experiments**. Replication capacities (RC) were determined by culturing the viruses in SupT1 cells in absence of inhibitor and monitoring p24 production[28]. Error bars indicate the standard deviation. Replication of NL4-3 is comparable to that of HXB2 (not shown).

To assess the impact of different PI resistant backgrounds on selection of bevirimat resistance, we performed multiple *in vitro *resistance selection studies with a set of ten different viruses. Two wild-type viruses (HXB2 and NL4-3), two viruses that harbored PI resistance associated mutations in the NC/p1cleavage site (but had wild-type proteases) and six viruses that had PI resistance mutations in the viral protease (Table 1) were studied. The broad range of replication capacities of these viruses (Figure 1) allowed us to investigate the impact of RC on selection of bevirimat resistance.

**Table 1:** Characteristics of the ten viruses that were used for the *in vitro *selection experiments

HIV-1 variant	Mutations compared to HXB2		PI resistance	
	
	Gag	Protease	LPV	ATV
HXB2	-	-	-	-

NL4-3	-	3I-37N	1	1

PR-1		3I-**20R**-35D-**36I**-**54V**-**63P**-**71V**-**82T**	12.2	5.6

PR-2	431V	3I-**10I**-**13V**-35D-**36I**-37D-**46I**-**54V**-55R-57K-**62V**-**63P**-**71T**-**82A**-**90M**-**93L**-95F	15.1	7.8

PR-3^#^	431V	3I-**10I**-**13V**-35D-**36I**-37D-**54V**-55R-57K-**62V**-**63P**-**71T**-**82A**-**90M**-**93L**-95F	> 120	> 120

PR-4^#^	431V	3I-**10I**-**13V**-35D-**36I**-37D-**46I**-55R-57K-**62V**-**63P**-**71T**-**82A**-**90M**-**93L**-95F	8.1	11.6

PR-5^#^	431V	3I-**10I**-**13V**-35D-**36I**-37D-**46I**-**54V**-55R-57K-**62V**-**63P**-**71T**-**90M**-**93L**-95F	10.8	8.9

PR-6^#^	431V	3I-**10I**-**13V**-35D-**36I**-37D-**46I**-**54V**-55R-57K-**62V**-**63P**-**71T**-**82A**-**93L**-95F	19.6	5.9

NC/p1	431V	-	2.6	1.3

NC/p1	436E-437T	-	4.7	3.3

HXB2 and NL4-3 are subtype B reference viruses. Mutations are as compared to HXB2. All amino acid differences in the viral protease are listed. All protease inhibitor (PI) resistance mutations, as defined by the International AIDS Society[37] are in bold. In addition, mutations in the NC/p1 cleavage site are listed. The CA/p2 cleavage site of the ten viruses was identical. PR-1 is clone 460.2 from Nijhuis et al. [21] and PR-2 through PR-6 are the B6 clones from Maarseveen et al. [30]. ^#^PR-3 - PR-6 are site directed mutants created from PR-2. In each of these clones one PI resistance mutation was reverted to wild-type, PR-3 - PR-6 lack PI resistance mutation 46I, 54V, 82A and 90M respectively. The NC/p1 variants only differ from HXB2 at the positions indicated in the table. The level of PI resistance was determined for these viruses against lopinavir (LPV) and atazanavir (ATV). PI resistance is expressed as fold change in EC_50 _compared to HXB2.

During the *in vitro *selection experiments, there were no major differences in the rate of virus propagation in the presence of bevirimat between wild-type viruses and viruses with PI resistance mutations in the viral protease. Regardless of their RC, all viruses reached full-blown cytopathic effect (CPE) in a comparable number of days during each serial passage. However, independent of their RC, viruses with NC/p1 CS mutations (without mutations in protease), showed delayed propagation. The delay was primarily the result of a relatively long first passage, with subsequent passages being similar in duration to those of the other viruses (see Additional file 1).

After five serial passages to a final concentration of 240 nM bevirimat, RNA was isolated from all viruses and full Gag and protease genes sequenced. In all cultures, mutations in or near the CA/p2 cleavage site were found clearly supporting the hypothesis that this is the main target region for bevirimat (Tables 2 and 3). Gag mutations outside this region were found only in a small number of isolates and appeared to be random. The protease gene was completely conserved in all viruses (data not shown).

**Table 2:** Mutations selected in viruses with wild-type proteases during the bevirimat *in vitro *selections

Wild-type proteases													
	**CA**					**p2**							

**Gag position**	**359**	**360**	**361**	**362**	**363**	**364**	**365**	**366**	**367**	**368**	**369**	**370**	**371**

**HXB2 aa**	**K**	**A**	**R**	**V**	**L**	**A**	**E**	**A**	**M**	**S**	**Q**	**V**	**T**

**WT** **HXB2** n = 10	**-**	**-**	**-**	**-**	**-**	**V**	**-**	**-**	**-**	**-**	**-**	**-**	**-**
	-	-	-	-	-	-	-	-	-	-	-	A	-
	
	-	-	-	-	-	**V**	-	-	-	-	-	-	-
	
	-	-	-	-	-	**V**	-	-	-	-	-	-	-
	
	-	-	-	**V/I**	-	-	-	-	-	-	-	-	-
	
	-	-	-	-	-	**V**	-	-	-	-	-	-	-
	
	-	-	-	-	-	**V**	-	-	-	-	-	-	-
	
	-	-	-	**V/I**	-	**A/V**	-	-	-	-	-	-	-
	
	-	-	-	-	-	**V**	-	-	-	-	-	-	-
	
	-	-	-	-	-	**V**	-	-	-	-	-	-	-

**WT** **NL4-3** n = 10	-	-	-	-	-	**A/V**	-	**A/V**	-	-	-	-	-
	
	-	-	-	-	-	**A/V**	-	-	-	-	-	V/A	-
	
	-	-	-	-	-	**V**	-	-	-	-	-	-	-
	
	-	-	-	-	-	**V**	-	-	-	-	-	-	-
	
	-	-	-	-	-	**V**	-	-	-	-	-	-	-
	
	-	-	-	-	-	**A/V**	-	-	-	-	-	-	-
	
	-	-	-	-	-	**V**	-	-	-	-	-	-	-
	
	-	-	-	-	-	**V**	-	-	-	-	-	-	-
	
	-	-	-	-	-	**V**	-	-	-	-	-	-	-
	
	-	-	-	-	-	**V**	-	-	-	-	-	-	-

**NC/p1** **431V** n = 4	-	-	-	-	-	**V**	-	-	-	-	-	-	-
	
	-	-	-	-	-	**V**	-	-	-	-	-	-	-
	
	-	-	-	-	-	**V**	-	-	-	-	-	-	-
	
	-	-	-	-	-	**V**	-	-	-	-	-	-	-

**NC/p1** **436E-437T** n = 4	-	-	-	-	-	**V**	-	-	-	-	-	-	-
	
	-	-	-	**V/I**	-	**A/V**	-	-	-	-	-	-	-
	
	-	-	-	-	-	**V**	-	-	-	-	-	-	-
	
	-	-	-	-	-	**V**	-	-	-	-	-	-	-

Schematic representation of the amino acid changes appearing in the CA/p2 region during bevirimat *in vitro *selection experiments with wild-type HIV-1 or NC/p1 mutants. *In vitro *selections with wild-type viruses (HXB2 and NL4-3) were performed 10 times, the NC/p1 variants 5 times (n = 4 because one culture was discontinued for each virus). Mutations that previously have been identified *in vitro *as bevirimat resistance mutations are indicated in bold. The QVT-polymorphisms that are associated with a reduced response to bevirimat *in vivo *are underlined. The actual CA/p2 cleavage site is between amino acids 363 and 364.

**Table 3:** Mutations selected in viruses with PI resistant proteases during the bevirimat *in vitro *selections

PI resistant proteases													
	**CA**					**p2**							

**Gag position**	**359**	**360**	**361**	**362**	**363**	**364**	**365**	**366**	**367**	**368**	**369**	**370**	**371**

**HXB2 aa**	**K**	**A**	**R**	**V**	**L**	**A**	**E**	**A**	**M**	**S**	**Q**	**V**	**T**

**PR-1** n = 5	-	-	-	-	-	**V**	-	-	-	-	-	-	-
	
	-	-	-	-	-	**A/V**	-	-	-	-	-	-	-
	
	-	-	-	**V/I**	**L/M**	-	-	-	-	*N*	-	A	-
	
	-	-	-	**V/I**	-	-	-	-	-	*S/N*	-	-	T/N
	
	-	-	-	-	-	**A/V**	-	-	-	-	-	-	T/N

**PR-2** n = 5	-	-	-	-	-	-	-	-	-	*S/N*	-	-	-
	
	-	-	-	-	-	-	-	-	-	-	-	V/A	-
	
	-	-	-	**V/I**	-	**A/V**	-	-	-	-	-	-	-
	
	-	-	-	**V/I**	-	-	-	-	-	-	-	-	-
	
	-	-	-	-	-	-	-	-	-	-	-	A	-

**PR-3** n = 5	-	-	-	**V/I**	-	-	-	-	-	*S/N*	-	-	-
	
	-	-	-	-	-	**A/V**	-	-	-	-	-	-	-
	
	-	-	-	-	-	**V**	-	-	-	-	-	-	-
	
	-	-	-	-	-	-	-	-	-	*S/N*	-	V/A	-
	
	-	-	-	**V/I**	-	-	-	-	-	-	-	-	-

**PR-4** n = 5	-	-	-	-	-	**A/V**	-	-	-	-	-	-	-
	
	-	-	-	**V/I**	-	-	-	-	-	-	-	V/A	-
	
	-	-	-	**V/I**	-	-	-	-	-	-	-	-	-
	
	-	-	-	**V/I**	-	-	-	-	-	*S/N*	-	-	-
	
	-	-	-	-	-	-	-	-	-	-	-	A	-

**PR-5** n = 5	-	-	-	**V/I**	-	-	-	-	-	-	-	-	-
	
	-	-	-	-	**L/M**	**A/V**	-	-	-	-	-	-	-
	
	-	-	-	**V/I**	-	-	-	-	-	-	-	V/A	-
	
	-	-	-	-	-	-	-	-	-	-	-	V/*L*	-
	
	-	-	-	-	-	**A/V**	-	-	-	*S/N*	-	-	-

**PR-6** n = 5	-	-	-	**V/I**	-	-	-	-	-	*S/N*	-	-	-
	
	-	-	-	**V/I**	-	**A/V**	-	-	-	*S/N*	-	-	-
	
	-	-	-	**I**	-	-	-	-	-	-	-	-	-
	
	-	-	-	**V/I**	-	-	-	-	-	-	-	V/A	-
	
	-	-	-	-	-	-	-	-	-	-	-	V/A	-

Schematic representation of the amino acid changes appearing in the CA/p2 region after bevirimat *in vitro *selection experiments with the PR-1 - PR-6 mutants. *In vitro *selections with the protease mutants were performed 5 times. Mutations that previously have been identified *in vitro *as bevirimat resistance mutations are indicated in bold. The QVT-polymorphisms that are associated with a reduced response to bevirimat *in vivo *are underlined. Previously unknown mutations are printed in italic type. The actual CA/p2 cleavage site is between amino acids 363 and 364.

Viruses with wild-type proteases (HXB2, NL4-3 and NC/p1 variants) selected for Gag mutation A364V in 26 of 28 cultures, with additional mutations observed in 4 of these 26: two cultures had V362I+A364V; one had A366V+A364V and another one V370A+A364V (Table 2). These combinations of mutations were thought to represent separate populations, with no viruses harboring two CA/p2 mutations on one genome. This was confirmed by clonal analysis of one culture containing multiple mutations (culture HXB2 #8; V362I+A364V, data not shown). In the two cultures where A364V was not selected, mutations V362I and V370A were observed respectively.

In contrast to viruses with WT proteases, viruses with PI resistant proteases (PR-1 - PR-6) showed a much more diverse resistance pattern (Table 3) with a significantly higher prevalence of mutations at position 362, 368 and in the QVT-motif (V370A/L and T371N, Table 4).

**Table 4:** Differences in mutations selected during the bevirimat *in *vitro selections

mutation	Wild-type proteases n (%)	PI resistant proteases n (%)	p-value
V362I	3/28 (10.7)	15/30 (50.0)	0.002

A364V	26/28 (92.9)	10/30 (33.3)	< 0.001

S368N	0/28 (0)	9/30 (30.0)	0.002

V370A	2/28 (7.1)	9/30 (30.0)	0.043

all QVT	2/28 (7.1)	12/30 (40.0)	0.005

The differences in mutations that were selected with viruses with either wild-type or PI resistant proteases during the bevirimat *in vitro *selections are listed. Absolute frequencies and the proportion of cultures harboring the mutations are given. *P*-values were determined using Fisher's exact test.

In summary, we identified 8 bevirimat resistance mutations at 7 different codons: V362I, L363M, A364V, A366V, S368N, V370A/L and T371N. Most of these mutations have been selected during *in vitro *selections in previous studies, or have already been associated *in vivo *with reduced bevirimat susceptibility, except for the mutation at position 368, which was considered a potential new resistance mutation. In all 58 isolates at least one of the following mutations was found: Gag V362I, A364V, S368N or V370A/L.

### Impact of PI resistance mutations on bevirimat resistance and viral replication

To characterize the different bevirimat resistance profiles observed in viruses with wild-type and PI resistant proteases, we investigated the impact of the four most frequently selected mutations on bevirimat susceptibility and viral replication in different genetic backgrounds. Therefore we introduced Gag mutations V362I, A364V, S368N or V370A by site-directed mutagenesis in the background of HXB2, PR-1 and PR-2. The bevirimat susceptibility and the relative replication capacity of these 12 viruses were determined.

In wild-type HXB2, mutations A364V and V370A conferred the highest level of resistance (Table 5). Both mutations resulted in a complete lack of inhibition even at 3000 nM (> 150-fold reduced susceptibility to bevirimat, Table 5 and Additional file 2, panels A and B). Mutations V362I and S368N resulted in low-level resistance (2.8-fold and 6.6-fold respectively). In the context of protease mutant PR-1, fold changes for the four site-directed mutants were almost identical to that of HXB2 (Table 5). However, the results were quite different for the mutations in the PR-2 background. Again, mutations A364V and V370A conferred > 150-fold resistance but, interestingly, mutations V362I and S368N, which demonstrated only low-level resistance in the background of HXB2 and PR-1, also resulted in the fully resistant phenotype when introduced in PR-2 (see Additional file 2, compare panels C and E with D and F). This revealed that the newly identified S368N mutation indeed is a bevirimat resistance mutation, which results in low-level resistance in a wild-type protease background but can give high-level resistance in the context of a mutated protease.

**Table 5:** Impact of PI resistance mutations on bevirimat resistance

Virus	Fold resistance bevirimat				
	
	-	V362I	A364V	S368N	V370A
**HXB2**	-	2.8	> 150	6.6	> 150

**PR-1 (20R-36I-54V-63P-71I-82T)**	0.6	2.1	> 150	6.0	> 150

**PR-2 (431V-10I-13V-36I-46I-54V-62V-63P-71T-82A-90M-93L)**	2.1	> 150	> 150	> 150	> 150

Levels of bevirimat resistance caused by single CA/p2 mutations in different protease backgrounds are given. Resistance is expressed as fold change in EC_50 _compared to HXB2. All numbers are averages of at least two separate experiments.

We also tested if the bevirimat resistance mutations affected PI (lopinavir and atazanavir) susceptibility. All site-directed mutants with the bevirimat resistance mutations in the HXB2 and PR-1 backgrounds were analyzed. None of these Gag mutations had a substantial effect on PI susceptibility; all changes in EC_50 _were below 2-fold (see Additional file 3). As an additional control, the susceptibility to lopinavir and atazanavir was tested for virus PR-2GagS368N. Compared to virus PR-2, fold changes in susceptibility were 1.7 and 1.1-fold respectively. Furthermore, the susceptibility of HXB2GagV370A to PIs tipranavir, saquinavir, nelfinavir, indinavir and the NRTI zidovudine was determined. Fold changes in EC_50 _compared to HXB2 were 0.9, 1.0, 1.8, 0.9 and 1.2-fold respectively.

**Figure 2:**
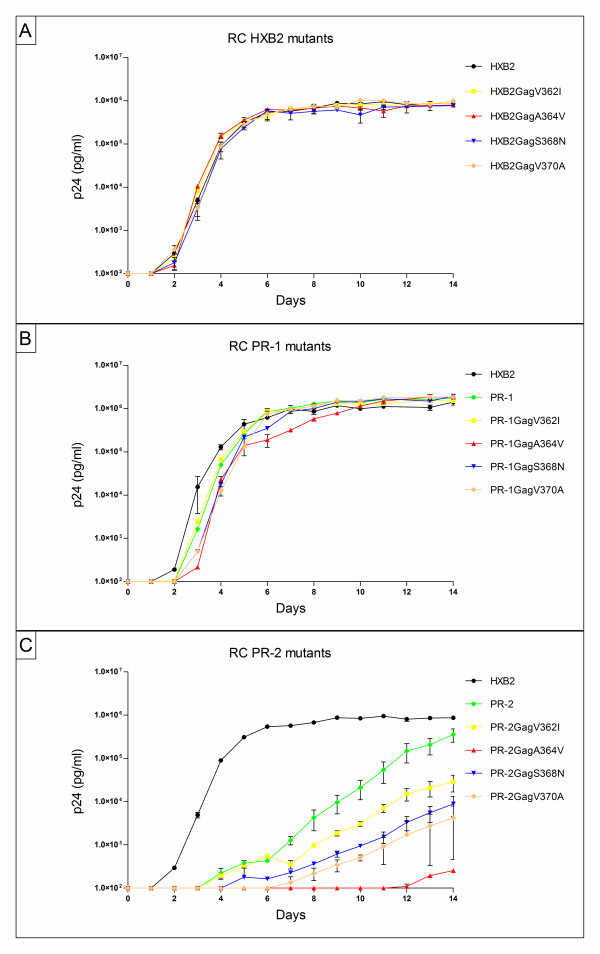
**Impact of bevirimat resistance mutations on viral replication in different genetic backgrounds**. Viruses were cultured in SupT1 cells in absence of inhibitor and p24 production was monitored for 14 days. All viruses were tested in duplicate. Error bars indicate the standard deviation. Replication curves of (A) the HXB2 site-directed mutants, (B) the PR-1 mutants and (C) the PR-2 mutants.

The relative RC of the 12 site-directed mutants was assayed by culturing virus in the absence of inhibitor for 14 days and monitoring p24 production. None of the four resistance mutations had an apparent effect on viral replication in the background of HXB2 wild-type virus (Figure 2A). Similarly, in PR-1, which had an RC comparable to that of HXB2, the introduction of any of the four bevirimat resistance mutations had little effect on replication. There was a slight delay in replication of viruses PR-1GagA364V, PR-1GagS368N and PR-1GagV370A but these differences were very small (one day) and the slopes of the curves and end-point replication were similar to those of the reference virus and other PR-1 strains (Figure 2B). In contrast, we observed large differences in RC for the mutations in the PR-2 background (Figure 2C). The parental virus (PR-2) already exhibited reduced replication compared to HXB2 wild-type virus and all four bevirimat resistance mutations further lowered the RC of the virus, to very different extents. Virus PR-2GagV362I displayed the highest replication capacity of the four site-directed mutants but replication was still substantially lower than that of PR-2. Mutations S368N and V370A had a more severe impact resulting in intermediate replication levels. Mutation A364V was highly detrimental in this background and reduced viral replication to a minimum.

### Effect of bevirimat resistance mutations on CA/p2 processing efficiencies

In order to characterize the differences in resistance levels conferred by bevirimat resistance mutations in different genetic backgrounds, we performed a biochemical analysis of the specific cleavage efficiencies. The effect of mutations V362I and A364V on CA/p2 processing was analyzed in the background of HXB2 and PR-2 proteases. Nonapeptides representing the WT CA/p2 cleavage site, or containing bevirimat resistance mutation V362I or A364V, were processed with either the HXB2 or the PR-2 protease enzyme. Although the absolute cleavage efficiency of PR-2 was lower compared to HXB2, the relative increase in processing caused by adding mutation V362I or A364V was approximately 50% greater for the PR-2 protease than for the HXB2 protease (Table 6). For both proteases, processing of the peptide with mutation A364V was one order of magnitude faster compared to V362I.

**Table 6:** CA/p2 processing efficiencies of the HXB2 and PR-2 proteases

Substrate	Relative substrate conversion		
	
	HXB2	PR-2	Ratio (PR-2/HXB2)
**WT**	1	1	-

**V362I**	0.87	1.3	1.49

**A364V**	7.6	11	1.45

Comparison of the CA/p2 processing efficiencies of the HXB2 and PR-2 protease enzymes. Three different nonapeptides representing the CA/p2 cleavage site were cleaved with either the HXB2 or the PR-2 protease: 1. wild-type (WT) KARVL↓AEANLe-NH_2_, 2. (V362I) KARIL↓AEANLe-NH_2 _and 3. (A364V) KARVL↓VEANLe-NH_2_. The bevirimat resistance mutations are underlined and the arrow indicates the actual junction. The cleavage efficiency of the WT substrate was set to 1, conversion of substrates with V362I or A364V was measured relative to the conversion of the WT substrate. The test was performed in triplicate.

## Discussion

Maturation inhibitors are an experimental class of antiretrovirals that prevent HIV-1 replication by targeting the structural proteins essential for particle maturation and thus formation of infectious virions. The target region for maturation inhibitors, Gag, is the same as the natural substrate of the viral protease. Co-evolution of protease and Gag during PI therapy[1,5,21-23] may, therefore, have consequences for the subsequent use of maturation inhibitors. We used bevirimat in a model system to study the impact of PI therapy on the development of resistance against maturation inhibitors. To date, no direct cross-resistance between PIs and bevirimat has been observed[14,20,24], but it is conceivable that reduced viral replication, as often caused by PI resistance mutations, influences the emergence of bevirimat resistance. Therefore, we wanted to include the effect of viral replication capacity on development of maturation inhibitor resistance in our studies. We chose viruses PR-1 through PR-6 for our resistance selection studies because of their broad range of replication capacities.

During *in vitro *selection, there were only small differences in rates of virus propagation in the consecutive passages between the wild-type and PR-1 - PR-6 viruses. We did not find a clear correlation between the rate of selection for bevirimat resistance and the viral replication capacity. The differences within the individual cultures from a particular molecular clone were often larger than the differences between the averages of the various clones. However, the viruses with mutations only in the NC/p1 cleavage site appeared to have a delayed emergence of bevirimat resistance. This delay cannot be explained by viral replication capacity since this was comparable for the 431V mutant and wild-type virus. A possible explanation is that altering both rate-limiting cleavage sites (CA/p2 and NC/p1) without having an adapted protease is unfavorable for the virus.

In all our *in vitro *selection cultures, mutations were selected in or slightly downstream from the CA/p2 cleavage site. We showed that PI resistance mutations have a substantial impact on the selection of bevirimat resistance: the resistance profiles were remarkably different for viruses with PI resistant proteases compared to wild-type proteases. Mutation A364V occurred most frequently and was associated with a completely resistant phenotype in all three protease backgrounds (HXB2, PR-1 and PR-2). This mutation had no effect on replication in an HXB2 background, which might explain the almost exclusive selection of A364V by viruses with a wild-type protease. We also observed selection of multiple QVT-mutations. Although these mutations are known to cause bevirimat resistance, until recently they had only been found as naturally occurring polymorphisms in clinical isolates demonstrating reduced bevirimat susceptibility[17,18,25]. Knapp and colleagues showed selection of QVT-mutations in a different experimental setup in which they used mixed, clinically derived gag-protease recombinant HIV-1 samples to select for bevirimat resistance[26]. We have now shown that QVT-mutations can also be selected by clonal strains and wild-type virus. However, they are much more often selected by viruses with a mutated protease, which is in line with our previous *in vivo *observations[19]. We also showed this to be the case for mutation V362I, which recently has been identified as a natural polymorphism that confers bevirimat resistance[27]. In addition, we identified a previously unknown bevirimat resistance mutation, S368N. This mutation was not found in any cultures with wild-type proteases, but appeared frequently in cultures with PI resistant proteases.

Our results indicate that the protease background determines the level of resistance and the impact on replication. When introduced into the PR-2 background, mutations V362I and S368N result in much higher levels of resistance than in backgrounds HXB2 or PR-1. High levels of bevirimat resistance for mutation V362I have also been observed in other genetic backgrounds [27]. A possible explanation for these observations is the difference in cleavage efficiencies of the Gag substrate by the viral protease. It has previously been reported that the level of bevirimat resistance is reduced in a PI resistant virus with a reduced Gag processing efficiency[20]. We show that processing of the CA/p2 cleavage site is accelerated by the presence of a bevirimat resistance mutation, which is likely to augment bevirimat resistance, parallel to what is observed for PI resistance[4,5]. Both proteases that were tested (HXB2 and PR-2) processed substrate with mutation A364V one order of magnitude more effectively than substrate with a V362I mutation. This might explain the high levels of bevirimat resistance conferred by A364V in all backgrounds. Furthermore, the relative increase in substrate conversion is greater in the context of the PR-2 protease compared to the HXB2 protease and we hypothesize that this relative increase in CA/p2 processing contributes to the enhanced bevirimat resistance levels observed for the PR-2GagV362I and PR-2GagS368N viruses.

## Conclusions

Like most new drug classes, maturation inhibitors are likely to be introduced as part of salvage therapy. The majority of patients requiring new therapeutic options will be infected with viruses that harbor multiple resistance mutations, most likely including PI resistance mutations. Therefore, it is essential to understand the consequences of prior treatment with PIs for the use of maturation inhibitors. Our data show that predicting treatment responses for maturation inhibitors might not be straightforward and that the complex interactions between protease and Gag have to be taken into account.

The development of new and more potent maturation inhibitors should therefore aim to overcome the issues encountered by the current drugs with virus containing the baseline polymorphisms found in the C-terminal Gag region (QVT-mutations) and, ideally, new maturation inhibitors would exhibit synergy with protease inhibitors. They should capitalize on the reduced processing often caused by PI resistance mutations in such a way that there is added value from the use of a maturation inhibitor in salvage therapy for PI experienced patients.

## Methods

### Viral and cell culture

#### Cells

293T cells were maintained in DMEM with L-glutamine (Lonza, Verviers, Belgium) supplemented with 10% fetal bovine serum (FBS; Sigma-Aldrich, Zwijndrecht, The Netherlands) and 10 μg/ml gentamicin (Invitrogen, Breda, The Netherlands). SupT1 and MT-2 cells were maintained in RPMI 1640 with L-glutamine (Lonza) supplemented with 10% FBS and 10 μg/ml gentamicin.

#### Recombinant virus panel

We selected a panel of ten different viruses for *in vitro *resistance selection studies (Table 1). Two wild-type viruses (HXB2 and NL4-3), six recombinant viruses with PI resistance mutations in the viral protease (PR-1 - PR-6) and two recombinant viruses without mutations in the viral protease but with PI resistance associated mutations in the Gag NC/p1 cleavage site (NC/p1 431V and NC/p1 436E-437T). PR-1 and PR-2 were gag-protease recombinant viruses from patient isolates that had acquired resistance mutations during long-term PI therapy and have different resistance profiles and replication capacities. PR-1 was selected because it displayed wild-type replication kinetics despite the presence of multiple PI resistance mutations. In contrast, like most PI resistant isolates, PR-2 had a slightly defective replication compared to wild-type (Figures 1 and 2C). PR-3 through to PR-6 are site directed mutants created from PR-2 in which in each of these clones one PI resistance mutation was reverted to wild-type. This resulted in dramatic changes in RC (Figure 1) while the mutations were very stable; they remained present and no additional protease mutations were acquired during long term culture in T cells in the absence of PIs [28]. These findings were consistent with other studies that described a significant effect on RC of a single mutation in the viral protease[29-31]. The NC/p1 variants had divergent replications capacities and both conferred low-level PI resistance in the absence of mutations in the viral protease. Fold changes against the commonly used PIs lopinavir and atazanavir are given in Table 1 for all variants.

*In vitro *selections with wild-type viruses HXB2 and NL4-3 were performed 10 times (10 parallel *in vitro *selections per virus), all other viruses 5 times. One culture of each NC/p1 variant was discontinued because of inadequate viral replication.

#### Transfections

Viruses were generated by transfecting 293T cells with 10 μg of plasmid DNA of the molecular clones using Lipofectamine 2000 reagent (Invitrogen) according to the manufacturer's protocol. Cell free virus was harvested 2 days after transfection. Infectious virus titer (TCID_50_) was determined by end-point dilution assays in MT-2 cells.

#### In vitro selections

Multiple *in vitro *selection experiments were started simultaneously for all viruses, 10 times with reference viruses HXB2 and NL4-3 and 5 times with all other viruses in. The *in vitro *resistance selections were started by infecting 2.0 × 10^6 ^SupT1 cells with virus at a multiplicity of infection (MOI) of 0.001. Bevirimat concentration in the initial cultures was 20 nM. Cultures were monitored daily for cytopathic effect (CPE) and twice a week half of the culture was replaced by fresh culture media supplemented with bevirimat. When full-blown CPE was observed, cell free virus was harvested. Subsequent passages were started by infecting 2.0 × 10^6 ^SupT1 cells with virus containing supernatant from the previous passage. Bevirimat concentration was raised in each passage to a final concentration of 240 nM in passage 5. After passage 5, HIV-1 RNA was isolated from all cultures for genotypic analysis.

### Genotypic analysis

#### Viral RNA extraction, amplification and sequencing

HIV-1 RNA was extracted using the Nuclisens Isolation kit (BioMérieux, Boxtel, The Netherlands). 100 μl of virus supernatant was added to 900 μl lysis buffer with 40 μl silica beads. After 10 minutes incubation, beads were washed twice in wash buffer, twice in 100% ethanol and once in acetone and subsequently air-dried. RNA was eluted at 56°C with 100 μl of 40 ng/μl poly-A RNA. Full Gag and protease genes were reversed transcribed and amplified in a single-step reaction using the Titan One Tube RT-PCR kit (Roche Diagnostics, Almere, The Netherlands). In a second PCR using the Expand High Fidelity kit (Roche) the amount of product was further enhanced. In the first PCR primers KVL 064 (5'-G TTG TGT TGT GAC TCT GGT AAC TAG AGA TCC CTC AGA-3'; 570-603)[32] and 3'prot-6 (5'-TTT TCA GGC CCA ATT TTT GAA ATT TT-3'; 2710-2685) were used. The second PCR was carried out with primers 5'-Anna (5'-ACT CGG CTT GCT GAA GCG CGC-3'; 696-716) and 3'prot-5 (5'-TGC TTT TAT TTT TTC TTC TGT CAA TGG CCA-3'; 2648-2619). Sequence analysis was performed with the BigDye Terminator v3.1 Cycle Sequencing Kit (Applied Biosystems, Foster City, CA, USA). Full Gag and protease sequences were obtained using a set of ten primers: GA1 (5'-GAC GCA GGA CTC GGC TTG CT-3'; 688-707), MArev-1 (5'-TGA TGT ACC ATT TGC CCC T-3'; 1223-1205), HXB2Gagfor[33], Sk38 (5'-ATA ATC CAC CTA TCC CAG TAG GAG AAA T-3'; 1544-1571), Sk39 (5'-TTT GGT CCT TGT CTT ATG TCC AGA ATG C-3'; 1658-1631), NCrev-1 (5'- TGT GCC CTT CTT TGC CAC AAT-3'; 1990-1970), 5'clea-4 (5'-ATA ATG ATG CAG AGA GG-3'; 1915-1931) and 3'CS1, PR2 and PR5 [34].

### Site-directed mutagenesis

In viruses HXB2, PR-1 and PR-2, Gag substitutions V362I, A364V, S368N and V370A were introduced by site-directed mutagenesis. Therefore PCR was performed on the respective plasmids using Vent_R _DNA polymerase (New England Biolabs, Ipswich, MA, USA) with primers 5'-Anna and 3'prot-6 and a third mutagenesis primer: GagV362 (5'-GGC AAG AAT TTT GGC TGA AGC AAT G-3';1866-1890), GagA364V (5'-GGC AAG AGT TTT GGT TGA AGC AAT G-3';1866-1890), GagS368N (5'-GGC TGA AGC AAT GAA CCA GGT AAC CA-3';1878-1903) or GagV370A (5'-GCA ATG AGC CAG GCA ACC AAT TC; 1885-1907).

The full Gag and protease PCR fragments were digested with restriction enzymes BssHII and MluNI. Digested fragments were then cloned into the previously described HXB2 reference vector CP-Wt[33] that also was digested with BssHII and MluNI. PCR product and vector (pHXB2ΔGagPR) were ligated using the Rapid DNA Ligation System (Promega Benelux, Leiden, The Netherlands) and subsequently transformed in competent cells.

### Phenotypic analysis

#### Drug susceptibility analysis

Drug susceptibility was determined by a multiple cycle cell-killing assay[35]. MT-2 cells (5 × 10^4 ^in 200 μl RPMI 10% FBS per well) were plated in 96-well microplates. Sample virus and reference virus were inoculated for five days on a single 96-well plate in the presence of threefold dilutions of bevirimat. Both sample virus and reference virus were inoculated at multiple MOIs to adjust for any differences in viral RC. Fold change (FC) values were calculated by dividing the mean 50% effective concentration (EC_50_) for a sample virus by that of the HXB2 reference strain. Fold changes are averages of at least two separate experiments.

#### Viral replication assay

For each viral clone the amount of p24 was determined by ELISA (Aalto Bioreagent, Dublin, Ireland). Replication capacity was determined by infecting 2.0 × 10^6 ^SupT1 cells (in duplicate) with an equivalent of 100 ng p24 of each virus. After 2 hours of incubation, cells were washed twice with fresh RPMI 1640 medium with L-glutamine and subsequently resuspended in 10 ml RPMI 1640 medium with L-glutamine supplemented with 10% FBS and gentamicin. Cultures were maintained in the absence of inhibitor for fourteen days and once daily 300 μl of cell-free virus supernatant was harvested for p24 analysis.

### CA/p2 processing efficiencies of the HXB2 and PR-2 protease enzymes

HXB2 and PR-2 proteases were over-expressed in *E. coli *and purified to homogeneity as described previously[36]. Briefly, *E. coli *BL21(DE3)RIL (Novagen, Darmstadt, Germany) were transfected by pET 24a plasmid coding for the corresponding enzyme. The insoluble recombinant protein, accumulated in the form of inclusion bodies, was isolated and solubilized in 67% (v/v) acetic acid. The recombinant proteases were refolded by diluting in a 25-fold excess of water and overnight dialysis against water at 4°C followed by overnight dialysis against 50 mM 2-(*N*-morpholino)ethanesulfonic acid (MES) pH 5.8, 10% (v/v) glycerol, 1 mM ethylenediaminetetraacetic acid (EDTA) and 0.05% (v/v) 2-mercaptoethanol. The proteases were purified by cation exchange chromatography using MonoS FPLC (Amersham Biosciences, Uppsala, Sweden). Purified enzymes were stored at -70°C. The proteolytic activities of these enzymes were tested with substrates derived from Gag wt, GagV362I and GagA364V, represented by the following nonapeptides: KARVL↓AEANLe-NH_2_, KARIL↓AEANLe-NH_2_, and KARVL↓VEANLe-NH_2 _as previously described. Substrates (200 μM) were incubated for 25 min with either HXB2 or PR-2 protease (75 nM) enzyme in 50 mM MES buffer (300 mM NaCl, pH 6.0) at 37°C. The arrow indicates the actual cleavage site. The cleavage reaction was stopped by adding concentrated formic acid. Enzymatic reaction mixtures were resolved in triplicates on a Zorbax SB-C_18 _reversed phase HPLC column (4.6 × 150 mm, particle size 1.8 μm, Agilent Technologies, USA).

## Competing interests

The authors declare that they have no competing interests.

## Authors' contributions

AF, NMvM and MN conceived and designed the study. AF, NMvM, JP, REMM and PJS performed the experiments. AF, NMvM, JP, REMM, PJS, JK and MN analyzed the data. AF, NMvM, JK and MN wrote the paper. All authors read and approved the final version of the manuscript.

## Supplementary Material

Additional file 1 **Virus propagation in the presence of increasing bevirimat concentrations**. The cumulative number of days until full blown CPE was observed is shown averaged for each variant. Click here for file

Additional file 2 **Impact of protease background on bevirimat resistance**. Fold increase in bevirimat EC_50 _caused by single CA/p2 mutations in different protease backgrounds. Click here for file

Additional file 3 **Impact of bevirimat resistance mutations on PI susceptibility**. The impact of the bevirimat resistance mutations on PI susceptibility is presented. Click here for file

## References

[B1] Mammano F Petit C Clavel F Resistance-associated loss of viral fitness in human immunodeficiency virus type 1: phenotypic analysis of protease and gag coevolution in protease inhibitor-treated patients J Virol 1998 72 7632 7637 9696866 10.1128/jvi.72.9.7632-7637.1998 PMC110025

[B2] Nijhuis M van Maarseveen NM Lastere S Schipper P Coakley E Glass B Rovenska M de Jong D Chappey C Goedegebuure IW Heilek-Snyder G Dulude D Cammack N Brakier-Gingras L Konvalinka J Parkin N Krausslich HG Brun-Vezinet F Boucher CA A novel substrate-based HIV-1 protease inhibitor drug resistance mechanism PLoS Med 2007 4 e36 10.1371/journal.pmed.0040036 17227139 PMC1769415

[B3] Verheyen J Litau E Sing T Daumer M Balduin M Oette M Fatkenheuer G Rockstroh JK Schuldenzucker U Hoffmann D Pfister H Kaiser R Compensatory mutations at the HIV cleavage sites p7/p1 and p1/p6-gag in therapy-naive and therapy-experienced patients Antivir Ther 2006 11 879 887 17302250

[B4] Nijhuis M van Maarseveen NM Verheyen J Boucher CA Novel mechanisms of HIV protease inhibitor resistance Curr Opin HIV AIDS 2008 3 627 632 10.1097/COH.0b013e3283136cd9 19373034

[B5] Dam E Quercia R Glass B Descamps D Launay O Duval X Krausslich HG Hance AJ Clavel F Gag mutations strongly contribute to HIV-1 resistance to protease inhibitors in highly drug-experienced patients besides compensating for fitness loss PLoS Pathog 2009 5 e1000345 10.1371/journal.ppat.1000345 19300491 PMC2652074

[B6] Kilgore NR Reddick M Zuiderhof M Stanley D Nitz T Bullock P Allaway GP Martin DE Characterization of PA1050040, a second-generation HIV-1 maturation inhibitor IAS 2007, 4th IAS Conference On HIV Pathogenesis, Treatment and Prevention, Sydney, Australia 2007 Abstract MOPDX05

[B7] Beelen AP Otto J Fidler M Sanguinetti E Smiley P Balch A Medlock M Jackson M Swabb EA Phase 1, Single Ascending Oral Dose Study of the Safety, Tolerability, and Pharmacokinetics of a Novel HIV-1 Maturation Inhibitor in HIV Negative Healthy Subjects Program & Abstracts of the 16th Conference on Retroviruses and Opportunistic Infections, Montreal, Canada 2009 Abstract 57021874603

[B8] Baichwal V Austin H Brown B McKinnon R Yager K Kumar V Gerrish D Anderson M Carlson R Anti-viral Characterization in vitro of a Novel Maturation Inhibitor, MPC-9055 Program & Abstracts of the 16th Conference on Retroviruses and Opportunistic Infections, Montreal, Canada 2009 Abstract 56121874603

[B9] Kumar V Gerrish D Hoarau C Yager K Austin H McKinnon R Brown B Baichwal V Papac D Bradford C Patton S Bulka K DeMie L Carlson R Next Generation Orally Bioavailable HIV-1 Maturation Inhibitors239th ACS National Meeting & Exposition, San Francisco, USA, March 2010 Abstract 1371

[B10] Blair WS Cao J Fok-Seang J Griffin P Isaacson J Jackson RL Murray E Patick AK Peng Q Perros M Pickford C Wu H Butler SL New small-molecule inhibitor class targeting human immunodeficiency virus type 1 virion maturation Antimicrob Agents Chemother 2009 53 5080 5087 10.1128/AAC.00759-09 19805571 PMC2786326

[B11] Kelly BN Kyere S Kinde I Tang C Howard BR Robinson H Sundquist WI Summers MF Hill CP Structure of the antiviral assembly inhibitor CAP-1 complex with the HIV-1 CA protein J Mol Biol 2007 373 355 366 10.1016/j.jmb.2007.07.070 17826792 PMC2066180

[B12] Braun K Frank M Pipkorn R Reed J Spring H Debus J Didinger B von der Lieth CW Wiessler M Waldeck W HIV-1 capsid assembly inhibitor (CAI) peptide: structural preferences and delivery into human embryonic lung cells and lymphocytes Int J Med Sci 2008 5 230 239 18695744 10.7150/ijms.5.230 PMC2500149

[B13] Titolo S Mercier JF Wardrop E Schwedler U Goudreau N Lemke C Faucher AM Yoakim C Simoneau B Sundquist WI Mason S Discovery of Potent HIV-1 CapsidAssembly Inhibitors Program & Abstracts of the 17th Conference on Retroviruses and Opportunistic Infections, San Francisco, USA 2010 Abstract 5021874603

[B14] Li F Goila-Gaur R Salzwedel K Kilgore NR Reddick M Matallana C Castillo A Zoumplis D Martin DE Orenstein JM Allaway GP Freed EO Wild CT PA-457: a potent HIV inhibitor that disrupts core condensation by targeting a late step in Gag processing Proc Natl Acad Sci USA 2003 100 13555 13560 10.1073/pnas.2234683100 14573704 PMC263852

[B15] Adamson CS Ablan SD Boeras I Goila-Gaur R Soheilian F Nagashima K Li F Salzwedel K Sakalian M Wild CT Freed EO In vitro resistance to the human immunodeficiency virus type 1 maturation inhibitor PA-457 (Bevirimat) J Virol 2006 80 10957 10971 10.1128/JVI.01369-06 16956950 PMC1642185

[B16] Keller PW Adamson CS Heymann JB Freed EO Steven AC HIV-1 maturation inhibitor bevirimat stabilizes the immature Gag lattice J Virol 2011 85 1420 1428 10.1128/JVI.01926-10 21106735 PMC3028903

[B17] McCallister S Lalezari J Richmond G Thompson M Harrigan PR Martin DE Salzwedel K Allaway GP HIV-1 Gag polymorphisms determine treatment response to bevirimat (PA-457) Antivir Ther 2008 13

[B18] Van Baelen K Salzwedel K Rondelez E Van Eygen V De Vos S Verheyen A Steegen K Verlinden Y Allaway GP Stuyver LJ Susceptibility of human immunodeficiency virus type 1 to the maturation inhibitor bevirimat is modulated by baseline polymorphisms in Gag spacer peptide 1 Antimicrob Agents Chemother 2009 53 2185 2188 10.1128/AAC.01650-08 19223634 PMC2681549

[B19] Verheyen J Verhofstede C Knops E Vandekerckhove L Fun A Brunen D Dauwe K Wensing AM Pfister H Kaiser R Nijhuis M High prevalence of bevirimat resistance mutations in protease inhibitor-resistant HIV isolates Aids 2009 24 669 673 10.1097/QAD.0b013e32833160fa 19926962

[B20] Adamson CS Waki K Ablan SD Salzwedel K Freed EO Impact of human immunodeficiency virus type 1 resistance to protease inhibitors on evolution of resistance to the maturation inhibitor bevirimat (PA-457) J Virol 2009 83 4884 4894 10.1128/JVI.02659-08 19279107 PMC2682084

[B21] Zhang YM Imamichi H Imamichi T Lane HC Falloon J Vasudevachari MB Salzman NP Drug resistance during indinavir therapy is caused by mutations in the protease gene and in its Gag substrate cleavage sites J Virol 1997 71 6662 6670 9261388 10.1128/jvi.71.9.6662-6670.1997 PMC191944

[B22] Nijhuis M Schuurman R de Jong D Erickson J Gustchina E Albert J Schipper P Gulnik S Boucher CA Increased fitness of drug resistant HIV-1 protease as a result of acquisition of compensatory mutations during suboptimal therapy Aids 1999 13 2349 2359 10.1097/00002030-199912030-00006 10597776

[B23] Cote HC Brumme ZL Harrigan PR Human immunodeficiency virus type 1 protease cleavage site mutations associated with protease inhibitor cross-resistance selected by indinavir, ritonavir, and/or saquinavir J Virol 2001 75 589 594 10.1128/JVI.75.2.589-594.2001 11134271 PMC113954

[B24] Zhou J Yuan X Dismuke D Forshey BM Lundquist C Lee KH Aiken C Chen CH Small-molecule inhibition of human immunodeficiency virus type 1 replication by specific targeting of the final step of virion maturation J Virol 2004 78 922 929 10.1128/JVI.78.2.922-929.2004 14694123 PMC368845

[B25] Adamson CS Sakalian M Salzwedel K Freed EO Polymorphisms in Gag spacer peptide 1 confer varying levels of resistance to the HIV- 1 maturation inhibitor bevirimat Retrovirology 2010 7 36 10.1186/1742-4690-7-36 20406463 PMC2873507

[B26] Knapp DJ Harrigan PR Poon AF Brumme ZL Brockman M Cheung PK In Vitro Selection of Clinically Relevant Bevirimat Resistance Mutations Revealed by "Deep" Sequencing of Serially Passaged, Quasispecies-Containing Recombinant HIV-1 J Clin Microbiol 2011 49 201 208 10.1128/JCM.01868-10 21084518 PMC3020451

[B27] Margot NA Gibbs CS Miller MD Phenotypic Susceptibility to Bevirimat in Isolates from HIV-1-Infected Patients without Prior Exposure to Bevirimat Antimicrob Agents Chemother 2010 54 2345 2353 10.1128/AAC.01784-09 20308382 PMC2876391

[B28] van Maarseveen NM Wensing AM de Jong D Taconis M Borleffs JC Boucher CA Nijhuis M Persistence of HIV-1 variants with multiple protease inhibitor (PI)-resistance mutations in the absence of PI therapy can be explained by compensatory fixation J Infect Dis 2007 195 399 409 10.1086/510533 17205479

[B29] Mammano F Trouplin V Zennou V Clavel F Retracing the evolutionary pathways of human immunodeficiency virus type 1 resistance to protease inhibitors: virus fitness in the absence and in the presence of drug J Virol 2000 74 8524 8531 10.1128/JVI.74.18.8524-8531.2000 10954553 PMC116364

[B30] Resch W Ziermann R Parkin N Gamarnik A Swanstrom R Nelfinavir-resistant, amprenavir-hypersusceptible strains of human immunodeficiency virus type 1 carrying an N88S mutation in protease have reduced infectivity, reduced replication capacity, and reduced fitness and process the Gag polyprotein precursor aberrantly J Virol 2002 76 8659 8666 10.1128/JVI.76.17.8659-8666.2002 12163585 PMC136408

[B31] Gonzalez LM Brindeiro RM Aguiar RS Pereira HS Abreu CM Soares MA Tanuri A Impact of nelfinavir resistance mutations on in vitro phenotype, fitness, and replication capacity of human immunodeficiency virus type 1 with subtype B and C proteases Antimicrob Agents Chemother 2004 48 3552 3555 10.1128/AAC.48.9.3552-3555.2004 15328124 PMC514783

[B32] Van Laethem K Schrooten Y Dedecker S Van Heeswijck L Deforche K Van Wijngaerden E Van Ranst M Vandamme AM A genotypic assay for the amplification and sequencing of gag and protease from diverse human immunodeficiency virus type 1 group M subtypes J Virol Methods 2006 132 181 186 10.1016/j.jviromet.2005.10.008 16271771

[B33] van Maarseveen NM Huigen MC de Jong D Smits AM Boucher CA Nijhuis M A novel real-time PCR assay to determine relative replication capacity for HIV-1 protease variants and/or reverse transcriptase variants J Virol Methods 2006 133 185 194 10.1016/j.jviromet.2005.11.008 16368153

[B34] van Maarseveen NM de Jong D Boucher CA Nijhuis M An increase in viral replicative capacity drives the evolution of protease inhibitor-resistant human immunodeficiency virus type 1 in the absence of drugs J Acquir Immune Defic Syndr 2006 42 162 168 10.1097/01.qai.0000219787.65915.56 16645546

[B35] Boucher CA Keulen W van Bommel T Nijhuis M de Jong D de Jong MD Schipper P Back NK Human immunodeficiency virus type 1 drug susceptibility determination by using recombinant viruses generated from patient sera tested in a cell-killing assay Antimicrob Agents Chemother 1996 40 2404 2409 8891152 10.1128/aac.40.10.2404 PMC163542

[B36] Kozisek M Saskova KG Rezacova P Brynda J van Maarseveen NM De Jong D Boucher CA Kagan RM Nijhuis M Konvalinka J Ninety-nine is not enough: molecular characterization of inhibitor-resistant human immunodeficiency virus type 1 protease mutants with insertions in the flap region J Virol 2008 82 5869 5878 10.1128/JVI.02325-07 18400858 PMC2395164

[B37] Johnson VA Brun-Vezinet F Clotet B Gunthard HF Kuritzkes DR Pillay D Schapiro JM Richman DD Update of the drug resistance mutations in HIV-1: December 2010 Top HIV Med 2010 18 156 163 21245516

