# Supplementary material for: HIV-1 protease inhibitor mutations affect the development of HIV-1 resistance to the maturation inhibitor bevirimat
Source: Retrovirology. 2011 Aug 24;8:70. doi: 10.1186/1742-4690-8-70 (PMC3184055; doi:10.1186/1742-4690-8-70)
Supplement: Additional file 1 — Virus propagation in the presence of increasing bevirimat concentrations. The cumulative number of days until full blown CPE was observed is shown averaged for each variant. [file 1742-4690-8-70-S1.PDF]

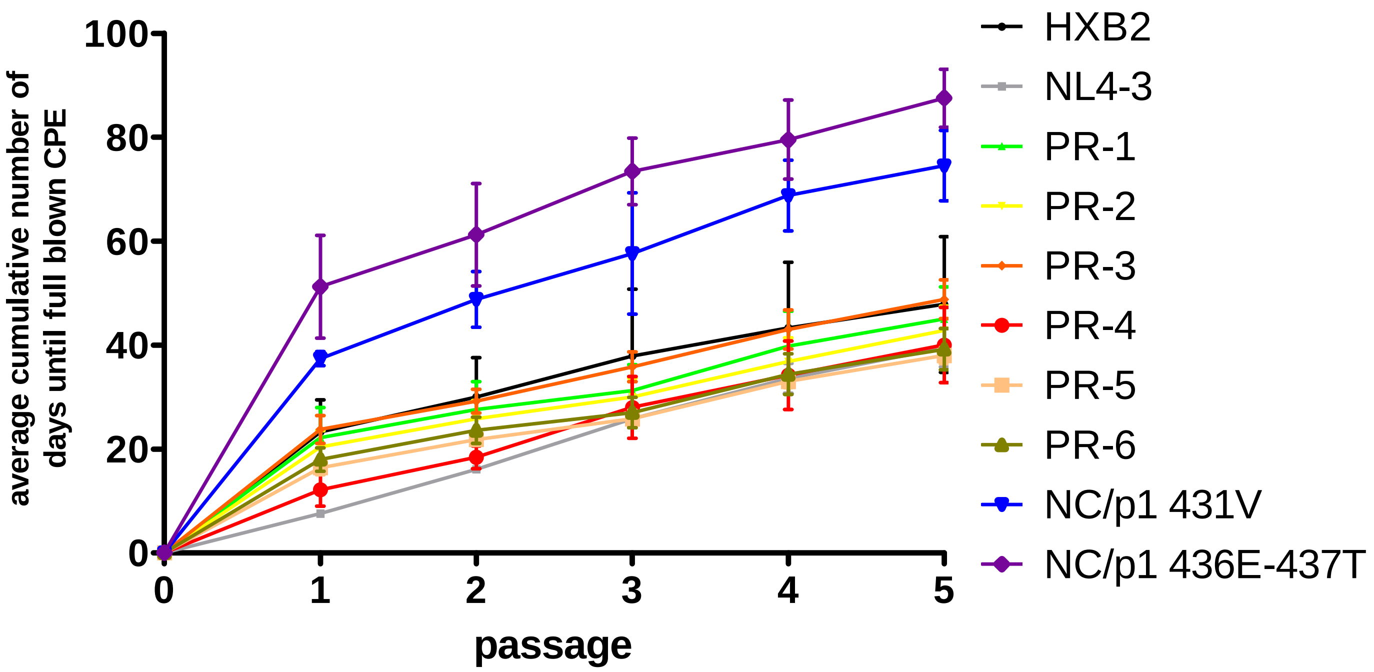


**Additional file 1 - Virus propagation in the presence of increasing bevirimat concentrations.** The cumulative number of days until full blown CPE was observed is shown averaged for each variant. SupT1 cells were infected with equal amounts of virus (multiplicity of infection of 0.001) and monitored for cytopathic effect (CPE). When full-blown CPE was reached, cell-free virus was harvested and used to infect the subsequent passage. The bevirimat concentration was increased from 20 nM in the first passage to 240 nM in the final passage. Error bars indicate the standard deviation.
