# Supplementary material for: HIV-1 protease inhibitor mutations affect the development of HIV-1 resistance to the maturation inhibitor bevirimat
Source: Retrovirology. 2011 Aug 24;8:70. doi: 10.1186/1742-4690-8-70 (PMC3184055; doi:10.1186/1742-4690-8-70)
Supplement: Additional file 2 — Impact of protease background on bevirimat resistance. Fold increase in bevirimat EC50 caused by single CA/p2 mutations in different protease backgrounds. [file 1742-4690-8-70-S2.PDF]

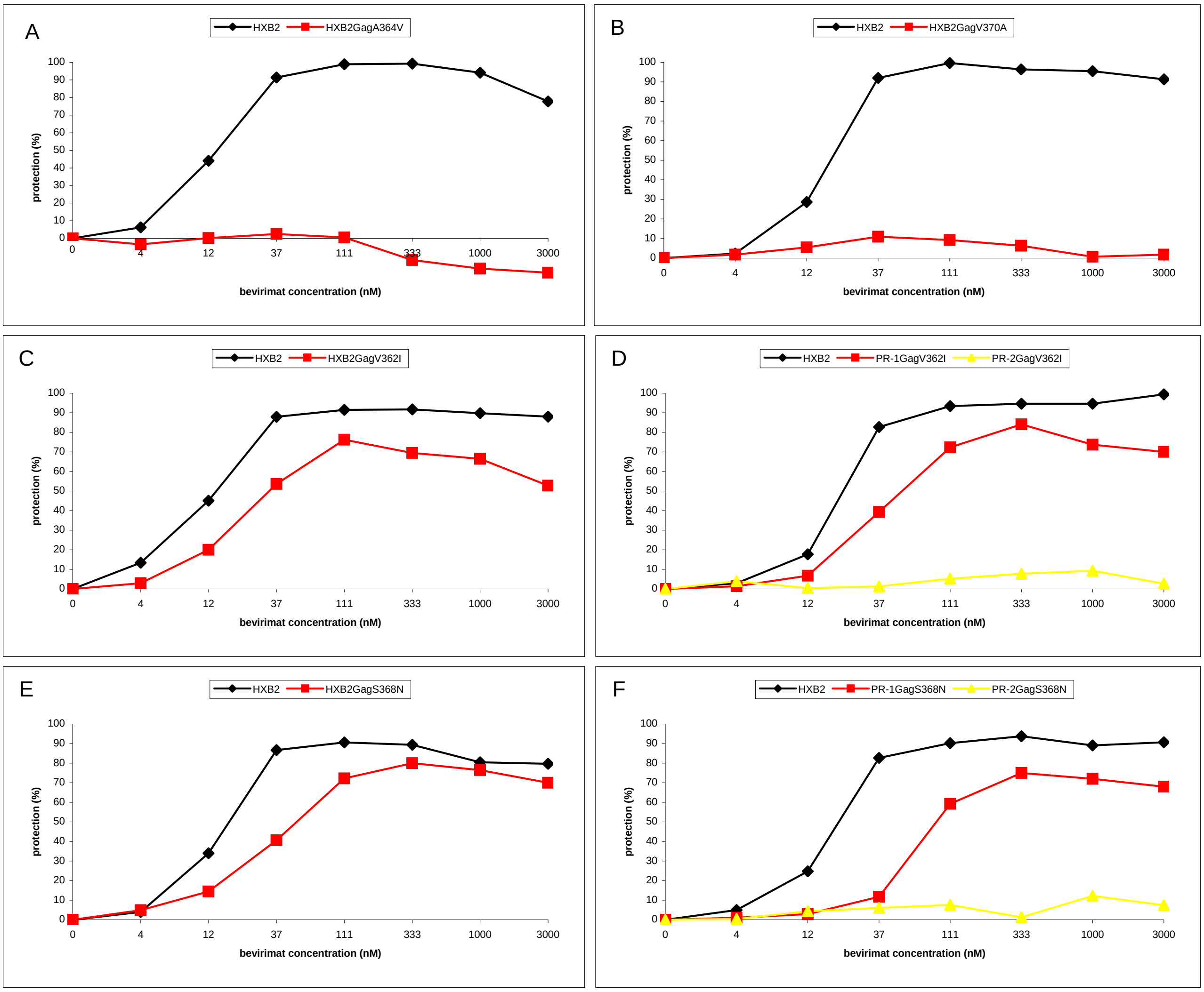

Additional file 2 - Impact of protease background on bevirimat resistance. Fold increase in bevirimat EC_{50} caused by single CA/p2 mutations in different protease backgrounds. All figures represent one of at least two separate experiments. (A-B) The susceptibility curves of site-directed mutants A364V and V370A in the HXB2 background are shown. (C-D) The susceptibility curves of virus with mutation V362I in wild-type vs. PR-1 and PR-2 backgrounds. (E-F) The same comparison is shown for viruses with mutation S368N.
