# Supplementary material for: HIV-1 protease inhibitor mutations affect the development of HIV-1 resistance to the maturation inhibitor bevirimat
Source: Retrovirology. 2011 Aug 24;8:70. doi: 10.1186/1742-4690-8-70 (PMC3184055; doi:10.1186/1742-4690-8-70)
Supplement: Additional file 3 — Impact of bevirimat resistance mutations on PI susceptibility. The impact of the bevirimat resistance mutations on PI susceptibility is presented. [file 1742-4690-8-70-S3.PDF]

Virus	Relative PI resistance							
	V362I		A364V		S368N		V370A	
	LPV	ATV	LPV	ATV	LPV	ATV	LPV	ATV
HXB2	1.2	1.4	0.6	0.8	0.8	1.4	1.1	1.7
PR-1	1.0	0.7	1.2	1.7	1.0	0.8	1.0	0.6

Additional file 3 – Impact of bevirimat resistance mutations on PI susceptibility. The impact of the bevirimat resistance mutations on protease inhibitor (PI) susceptibility is presented. Fold changes were determined for the two commonly used PIs lopinavir (LPV) and atazanavir (ATV). The change in EC_{50} is expressed relative to its parental virus.
